# Myosteatosis and Sarcopenic Obesity in Men Receiving Androgen Deprivation Therapy for Prostate Cancer: Rationale for Mechanism-Driven Multimodal Intervention

**DOI:** 10.3390/cancers18081276

**Published:** 2026-04-17

**Authors:** Nagi B. Kumar, Nathan Parker, Jingsong Zhang, Julio Pow-Sang, Jong Y. Park, Michael J. Schell

**Affiliations:** 1 Cancer Epidemiology Program, Moffitt Cancer Center and Research Institute, Tampa, FL 33612, USA; jong.park@moffitt.org; 2 Genitourinary Oncology Program, Moffitt Cancer Center and Research Institute, Tampa, FL 33612, USA; jingsong.zhang@moffitt.org (J.Z.); julio.powsang@moffitt.org (J.P.-S.); 3 Health Outcomes and Behavior Program, Moffitt Cancer Center and Research Institute, Tampa, FL 33612, USA; nathan.parker@moffitt.org; 4 Biostatistics and Bioinformatics Program, Moffitt Cancer Center and Research Institute, Tampa, FL 33612, USA; michael.schell@moffitt.org

**Keywords:** prostate cancer, androgen deprivation therapy, sarcopenic obesity, myosteatosis, muscle quality, metabolic abnormalities, inflammatory markers, multimodal intervention

## Abstract

**Background**: Androgen deprivation therapy (ADT) is widely used in the management of prostate cancer (PCa) and remains a cornerstone of treatment across multiple disease settings. Although ADT contributes substantially to disease control, it also induces significant adverse metabolic and body composition changes. These alterations include loss of lean mass, increased fat mass, and deterioration in muscle quality, together contributing to a clinical phenotype consistent with sarcopenic obesity (SO). Importantly, ADT-induced SO is characterized not only by reductions in skeletal muscle mass but also by impaired muscle quality, particularly the fatty infiltration of skeletal muscle, or myosteatosis, an underrecognized but defining feature of this syndrome. **Methods**: This narrative review examines current evidence regarding interventions aimed at mitigating sarcopenic obesity in men treated with ADT for prostate cancer, identifies key gaps in the literature, and proposes a mechanism-driven path forward for intervention development. **Results**: Several exercise- and nutrition-based interventions have been evaluated in men receiving ADT and demonstrate improvements in selected outcomes such as muscle strength, body composition, and metabolic parameters. However, most studies have been limited by small sample sizes, short intervention durations, and a focus on isolated intervention components. Importantly, muscle quality and intramuscular fat infiltration (myosteatosis), a central component of sarcopenic obesity, have rarely been incorporated as biomarkers or endpoints in intervention trials targeting men receiving ADT. **Conclusions**: Future interventions designed to mitigate SO and its associated metabolic abnormalities should evaluate comprehensive, bundled strategies initiated early during ADT and sustained long enough to capture clinically meaningful changes. Outcomes should include biomarkers of muscle mass, strength, and quality, including imaging-based measures of myosteatosis, along with metabolic syndrome markers, inflammatory mediators, functional outcomes, adherence, and quality of life. These changes should evaluate the correlation with underlying biological mechanisms such as NF-κB signaling and pro-inflammatory cytokines. Such data may inform future phase III trials and ultimately support clinical strategies to mitigate ADT-related sarcopenic obesity and its downstream cardiometabolic and oncologic consequences.

## Simple Summary

Androgen deprivation therapy (ADT) is a cornerstone of prostate cancer treatment but is associated with significant adverse effects on body composition and metabolic health. These include loss of skeletal muscle, increased fat mass, and development of sarcopenic obesity, a condition linked to functional decline, metabolic syndrome, and potentially adverse oncologic outcomes. An important and underrecognized component of this phenotype is myosteatosis, defined as the fatty infiltration of skeletal muscle, which reflects impaired muscle quality and underlying metabolic dysfunction. This structured narrative review synthesizes current evidence on interventions aimed at mitigating sarcopenic obesity in men receiving ADT. While exercise and nutrition-based strategies have shown benefits in improving muscle strength and body composition, most studies are limited by small sample sizes, short durations, and the lack of assessment of muscle quality. Notably, myosteatosis-specific endpoints remain rarely incorporated in clinical trials of men receiving ADT. We propose a mechanism-driven framework in which ADT-induced metabolic and inflammatory changes promote myosteatosis, which in turn, contributes to sarcopenic obesity and associated clinical outcomes. Future interventions should incorporate multimodal strategies and include imaging-based measures of muscle quality to better guide clinical management and improve survivorship outcomes.

## 1. Introduction

In 2026, an estimated 333,830 new cases of prostate cancer will be diagnosed in the United States, and 36,320 men will die from the disease [1]. Because prostate cancer is androgen dependent [2], androgen deprivation therapy (ADT) has become a mainstay of treatment for men with metastatic disease [3] and is also used in conjunction with radiotherapy for selected patients with localized or locally advanced disease [4]. It is estimated that more than 45% of men with prostate cancer receive ADT during the course of treatment [3].

ADT includes several classes of agents, including luteinizing hormone-releasing hormone agonists and antagonists such as leuprolide, goserelin, and degarelix; antiandrogens such as bicalutamide and flutamide; androgen synthesis inhibitors such as abiraterone; and other agents such as ketoconazole. The duration of treatment varies by disease risk group, treatment intent, and concomitant therapies, ranging from 4 to 6 months to several years. Men with high-risk disease may receive long-term ADT for 18–36 months, whereas those with intermediate-risk disease may receive shorter courses of 4–6 months. Men with metastatic disease may remain on ADT indefinitely [5].

Although ADT has improved disease control and survival, treatment-related adverse effects continue to compromise long-term health and quality of survival. The hormonal shifts associated with ADT induce substantial changes in body composition and metabolism, including increased fat mass, reduced lean body mass, altered lipid profiles, hyperglycemia, decreased insulin sensitivity, osteoporosis, and metabolic syndrome [5,6,7,8,9,10]. These changes may culminate in a clinical phenotype consistent with sarcopenic obesity (SO), characterized by the coexistence of sarcopenia and excess adiposity [6,7,11,12,13]. ADT-induced testosterone depletion contributes to reduced muscle protein synthesis and increased muscle catabolism, while also promoting visceral adiposity and metabolic dysfunction [5,7,8,9].

Beyond reductions in muscle mass alone, a defining and increasingly recognized feature of sarcopenic obesity is impaired muscle quality, particularly the accumulation of fat within skeletal muscle, termed myosteatosis [11,12,14,15]. Sarcopenia is defined by reduced skeletal muscle mass and/or function; myosteatosis reflects impaired muscle quality characterized by fatty infiltration and reduced radiodensity; and sarcopenic obesity represents the coexistence of excess adiposity with diminished muscle mass and/or quality. These related but distinct phenotypes are quantified using imaging-based metrics such as skeletal muscle index (SMI), skeletal muscle radiodensity (SMD), and measures of inter- or intramuscular adipose tissue (IMAT). Myosteatosis, on the other hand, reflects ectopic lipid deposition within and between muscle fibers and is associated with reduced muscle strength, impaired metabolic function, insulin resistance, and systemic inflammation [14,16,17]. Emerging evidence suggests that myosteatosis may represent one of the most clinically informative manifestations of sarcopenic obesity because it captures deterioration in muscle quality rather than quantity alone [11,12,14] (Table 1). Although myosteatosis has been increasingly associated with adverse clinical outcomes, including reduced survival, increased treatment toxicity, and poorer functional status across multiple cancer populations, prospective and ADT-specific data remain limited [7,11,12].

The adverse effects of SO in men receiving ADT extend beyond body composition alone. Compared with sarcopenia in isolation, SO appears to exacerbate metabolic dysregulation and has been associated with cardiovascular disease, insulin resistance, and diabetes mellitus [6,11,16,18,19,20,21]. These metabolic derangements may also adversely influence cancer outcomes, including biochemical recurrence, development of castration-resistant disease, metastatic progression, and prostate-cancer-specific as well as all-cause mortality [8,20,22,23]. These risks may be compounded by age-related declines in muscle mass, bone mineral density, and metabolic resilience [13].

Consensus groups have emphasized that heterogeneity in definitions continues to limit comparability across studies and slow clinical translation [12]. Importantly, reliance on body mass index alone is inadequate, as it does not capture body composition, muscle function, or muscle quality, all of which are central to SO [12,13]. Recent work has highlighted myosteatosis as an emerging biomarker of cancer prognosis across multiple malignancies. In men receiving androgen deprivation therapy, this phenotype may represent a central manifestation of treatment-related metabolic toxicity. We therefore propose that myosteatosis should be considered a key biomarker of ADT-induced sarcopenic obesity [15], linking metabolic dysfunction, inflammation, and skeletal muscle deterioration in this population. Despite growing recognition of myosteatosis as a clinically relevant muscle phenotype, substantial gaps remain, including the lack of standardized definitions and imaging thresholds, limited mechanistic studies in human cancer populations, and a predominance of retrospective data. Importantly, myosteatosis remains largely unaddressed as a therapeutic target in oncology trials. Given its potential reversibility and early emergence in the disease and treatment trajectory, there is a critical need to establish myosteatosis as a measurable and actionable biomarker to guide intervention strategies. This narrative review synthesizes current evidence on myosteatosis in oncology, with a focus on its definition, biological underpinnings, clinical relevance, and translational potential as an early and modifiable biomarker of prostate cancer prognosis.

## 2. Materials and Methods

This manuscript was developed as a structured narrative review aimed at integrating current clinical, imaging, and mechanistic evidence related to androgen deprivation therapy (ADT), myosteatosis, and sarcopenic obesity. Relevant literature was identified through targeted searches of PubMed/MEDLINE, Embase, Scopus, and Web of Science, supplemented by citation tracking and manual screening of reference lists from key articles. The search covered studies published from January 2000 through December 2025, reflecting the period during which imaging-based characterization of muscle quality and myosteatosis emerged in oncology research. Search terms included combinations of Medical Subject Headings (MeSH) and keywords related to “androgen deprivation therapy,” “sarcopenia,” “obesity,” “myosteatosis,” “muscle quality,” AND “prostate cancer,” “cancer”, “oncology,” “survival,” “treatment toxicity,” “metabolic abnormalities,” and “body composition.”

Studies were included if they: (i) involved adult cancer populations, particularly prostate cancer patients receiving ADT; (ii) evaluated body composition, muscle quality (e.g., myosteatosis, skeletal muscle radiodensity, or intramuscular adipose tissue), or related metabolic outcomes; and/or (iii) provided clinical, translational, or mechanistic insights relevant to sarcopenic obesity or myosteatosis. Eligible study designs included observational cohort studies, retrospective imaging analyses, interventional studies, and mechanistic investigations.

Studies were excluded if they: (i) were conducted exclusively in non-cancer populations without clear mechanistic relevance; (ii) evaluated sarcopenia without assessment of muscle quality or fat infiltration; (iii) were conference abstracts lacking sufficient methodological detail; or (iv) were non-English publications.

Titles and abstracts were screened for relevance, followed by full-text reviews of eligible articles. A flow diagram summarizing the identification, screening, eligibility, and inclusion of studies is provided to enhance transparency of the study selection process.

Evidence was synthesized qualitatively using a structured narrative approach, with findings grouped by cancer type, imaging modality, and outcome domain (clinical, metabolic, and functional). Given substantial heterogeneity in study design, definitions of myosteatosis, and outcome reporting, formal meta-analysis was not performed.

As this review was narrative in nature, formal risk-of-bias scoring was not conducted. However, interpretation of findings considered study design, sample size, methodological rigor, imaging standardization, adjustment for confounding variables, and consistency across independent cohorts.

This approach was selected to allow integration of heterogeneous evidence spanning epidemiologic, imaging, and mechanistic domains, which are not readily amenable to quantitative synthesis, while still maintaining transparency in study identification and selection.

**Figure 1:**
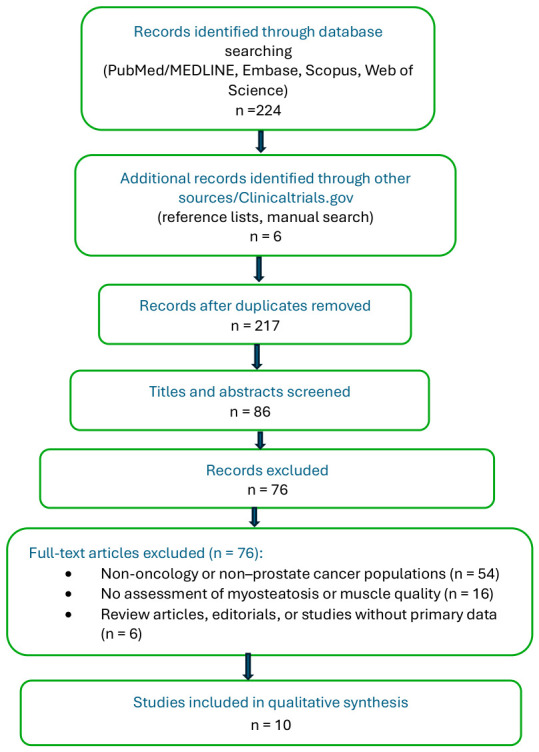
Study selection flow diagram. Flow diagram summarizing the identification, screening, eligibility assessment, and inclusion of studies in this structured narrative review. Records were identified through targeted database searches (PubMed/MEDLINE, Embase, Scopus, and Web of Science) and supplemented by manual review of reference lists. After duplicate removal, titles and abstracts were screened for relevance, followed by full-text reviews based on predefined inclusion and exclusion criteria. Reasons for exclusion at the full-text stage are provided. Studies meeting eligibility criteria were included in the qualitative synthesis to inform the conceptual framework and evidence synthesis presented.

A flow diagram summarizing the identification, screening, eligibility assessment, and inclusion of studies in this structured narrative review (Figure 1). Records were identified through targeted database searches (PubMed/MEDLINE, Embase, Scopus, and Web of Science) and supplemented by manual review of reference lists. After duplicate removal, titles and abstracts were screened for relevance, followed by full-text reviews based on predefined inclusion and exclusion criteria. Reasons for exclusion at the full-text stage are provided. Studies meeting eligibility criteria were included in the qualitative synthesis to inform the conceptual framework and evidence synthesis presented. A subset of studies specifically evaluating intervention strategies in men receiving ADT is summarized separately (Table 2), reflecting the limited number of trials incorporating comprehensive muscle quality endpoints.

## 3. Results

### 3.1. Skeletal Muscle-Specific Mechanisms Underlying Myosteatosis

The biological mechanisms underlying sarcopenic obesity (SO) in men receiving chronic androgen deprivation therapy (ADT) are thought to involve a complex interplay of inflammatory and metabolic signaling pathways that converge at the level of skeletal muscle. Experimental evidence suggests that activation of the ubiquitin–proteasome pathway, mediated in part through the upregulation of NF-κB, is associated with and likely contributes to skeletal muscle degradation and is stimulated by pro-inflammatory cytokines, including TNF-α and IL-6 [28,29,30,31,32,33]. These pathways, long implicated in muscle wasting and cachexia, are also implicated in the development of impaired muscle quality in the context of ADT.

Myosteatosis is increasingly recognized as a distinct pathological alteration in skeletal muscle characterized by ectopic lipid accumulation within muscle tissue, rather than solely a body composition phenotype. At the cellular level, increased deposition of intramyocellular and intermuscular adipose tissue contributes to reduced skeletal muscle radiodensity and disruption of contractile architecture. These changes have been associated with mitochondrial dysfunction, including reduced oxidative capacity and impaired lipid oxidation, which may further promote lipid accumulation within muscle fibers [34,35].

In parallel, local pro-inflammatory signaling within skeletal muscle—particularly NF-κB activation—drives increased expression of cytokines such as IL-6 and TNF-α, contributing to anabolic resistance and impaired muscle protein synthesis. Activation of proteolytic systems, including the ubiquitin–proteasome pathway, further accelerates muscle protein degradation. Additionally, fibro-adipogenic progenitor (FAP) cells may undergo lineage shifts favoring adipogenesis and fibrosis, reinforcing fat infiltration and structural remodeling of skeletal muscle [36].

Emerging evidence also highlights the role of the transforming growth factor-β (TGF-β) superfamily in regulating skeletal muscle homeostasis. Key ligands within this pathway, including myostatin and activins, function as important negative regulators of muscle mass through activation of activin receptor type II (ActRII) signaling, leading to SMAD2/3 phosphorylation and suppression of myogenic differentiation [37,38,39]. Upregulation of this pathway has been implicated in cancer-associated muscle wasting and may contribute to the development of myosteatosis through inhibition of muscle regeneration and promotion of fibro-adipogenic progenitor activity.

Importantly, crosstalk between inflammatory cytokines (e.g., IL-6, TNF-α) and TGF-β signaling pathways may amplify catabolic signaling, further promoting muscle protein degradation via ubiquitin–proteasome and autophagy–lysosome pathways. These integrated signaling networks suggest that myosteatosis reflects not only lipid infiltration but also impaired regenerative capacity and persistent catabolic activation within skeletal muscles.

Studies have demonstrated that selective exposure of the liver to orally administered testosterone reduced protein oxidation to the same extent as systemic (transdermal) testosterone administration [40,41]. On Inversely, suppression of testosterone to castrate levels during ADT has been shown to impact the hepatic urea cycle, resulting in greater nitrogen losses contributing to muscle catabolism [42]. Thus, increased hepatic urea production and whole-body protein oxidation have been identified as contributors to net protein loss in ADT-treated patients, including implications for nitrogen balance and interpretation of protein metabolism endpoints.

Collectively, these muscle-specific alterations support the characterization of myosteatosis as a distinct phenotype of impaired muscle quality, characterized by lipid accumulation, reduced radiodensity, and compromised metabolic and functional capacity.

### 3.2. Systemic Endocrine and Inflammatory Drivers of Myosteatosis in ADT

Beyond muscle-intrinsic mechanisms, androgen deprivation therapy (ADT) induces systemic endocrine and metabolic alterations that may further drive the development of myosteatosis. ADT-associated hypogonadism is associated with increased adiposity, particularly visceral fat accumulation, along with insulin resistance, dyslipidemia, and chronic low-grade inflammation [9,13,43]. These alterations may favor ectopic lipid deposition in peripheral tissues, including skeletal muscles.

Insulin resistance is associated with impaired glucose uptake and substrate utilization in the skeletal muscle, while elevated circulating free fatty acids may enhance lipid delivery and storage within muscle fibers. Concurrently, systemic inflammation—characterized by increased levels of IL-6 and TNF-α—may further exacerbate muscle catabolism and metabolic dysfunction. Hormonal alterations, including reduced testosterone and dysregulation of adipokines such as leptin and adiponectin, disrupt muscle–adipose crosstalk and promote anabolic resistance.

Androgen receptor (AR) signaling plays an important role in maintaining skeletal muscle homeostasis by promoting myogenic differentiation and suppressing adipogenic lineage commitment [17,44]. In the setting of androgen deprivation therapy, loss of AR signaling may disrupt mesenchymal stem cell fate decisions, shifting differentiation from myogenic toward adipogenic pathways. This lineage reprogramming is mediated, in part, through altered regulation of key transcription factors, including decreased MyoD and myogenin expression and increased activation of adipogenic regulators such as PPARγ and C/EBPα [17,44]. These processes provide a plausible mechanistic basis for the concurrent development of muscle loss and intramuscular fat accumulation observed in men receiving ADT.

In parallel, fibro-adipogenic progenitors (FAPs), which expand in response to injury and inflammation, may preferentially differentiate into adipocytes under conditions of hormonal deprivation and metabolic stress, further contributing to intramuscular fat deposition [45]. These findings suggest that myosteatosis may reflect active remodeling of muscle tissue rather than being a purely passive process.

**Figure 2:**
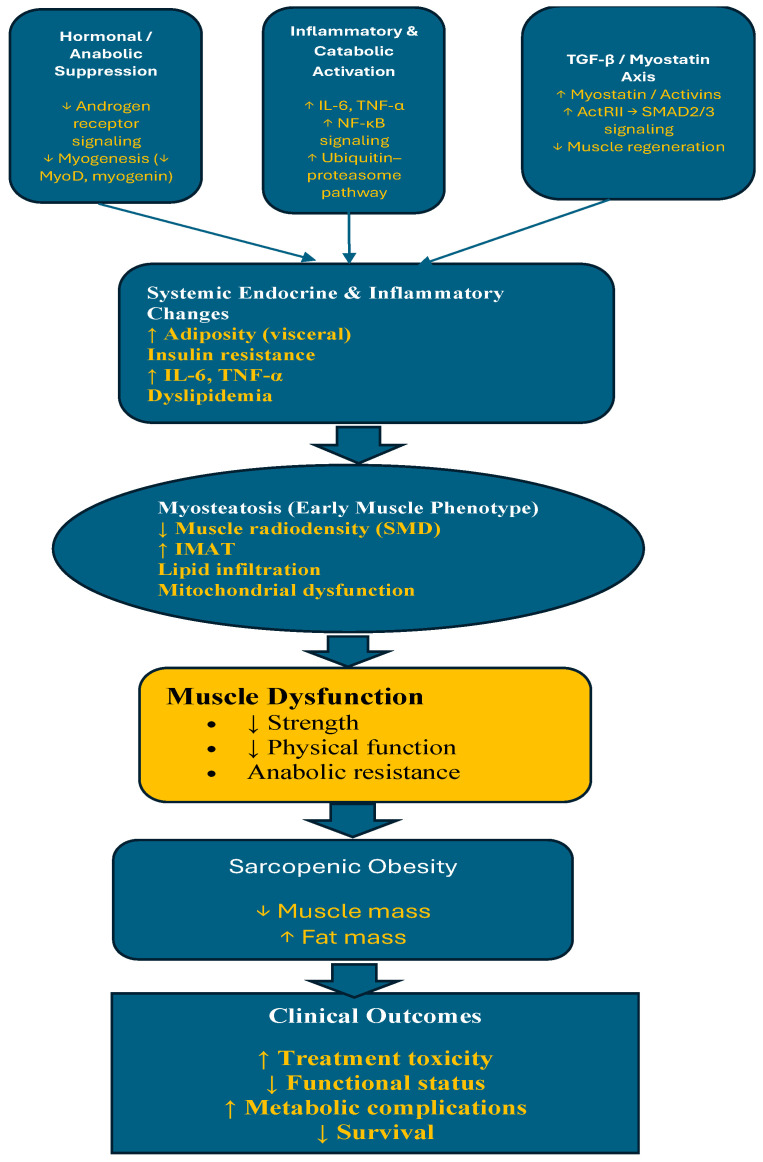
Conceptual framework linking androgen deprivation therapy (ADT) to myosteatosis, sarcopenic obesity, and potential intervention targets. ADT-induced hypogonadism promotes systemic endocrine and inflammatory alterations, including increased adiposity, insulin resistance, and chronic low-grade inflammation. These changes drive ectopic lipid deposition in skeletal muscle, resulting in myosteatosis characterized by reduced muscle radiodensity and impaired muscle function. Declines in muscle radiodensity may represent an early alteration preceding overt muscle loss. Progression of these processes contributes to sarcopenic obesity and adverse clinical outcomes. Multimodal interventions may target these pathways to improve muscle quality and metabolic health.

Together, these systemic perturbations are proposed to converge on skeletal muscle to promote lipid infiltration, reduced muscle radiodensity, and functional decline. The integration of these processes contributes to the development of sarcopenic obesity, linking systemic endocrine and inflammatory dysregulation with muscle-specific deterioration. These interconnected pathways provide the biological basis for the conceptual framework presented in Figure 2.

This conceptual framework generates several testable hypotheses. First, initiation of ADT will be associated with early declines in skeletal muscle radiodensity (SMD), reflecting increased intramuscular lipid infiltration and preceding measurable reductions in muscle mass. Second, changes in myosteatosis will correlate more strongly with metabolic dysregulation (e.g., insulin resistance and inflammatory cytokines) and clinical outcomes than changes in muscle mass alone. Third, multimodal interventions targeting inflammation, metabolic dysfunction, and muscle remodeling will preferentially improve muscle quality (SMD, IMAT) prior to detectable gains in lean mass. Finally, integrating imaging-based measures of myosteatosis with circulating biomarkers may enhance early risk stratification and identify patients most likely to benefit from targeted interventions. Collectively, this framework positions myosteatosis as both a clinically relevant biomarker and a potential therapeutic target in the prevention of ADT-related metabolic toxicity.

### 3.3. Current Strategies to Manage Sarcopenia, Obesity, and Other Metabolic Consequences of ADT

Although SO is well characterized in aging populations, it remains insufficiently defined in men with prostate cancer receiving ADT [7,12,13]. By contrast, several individual adverse effects of ADT, including obesity, sarcopenia, loss of bone mineral density, and vasomotor symptoms, are well documented [36,46,47,48,49,50]. Standard supportive therapies are therefore commonly used to manage selected toxicities. ADT has been shown to reduce bone mineral density measured by dual-energy X-ray absorptiometry. Bone mineral density has been reported to decline by approximately 2.4% in the first year and 7.6% over two years, with a reported range of 2.5–17.0% [46], and by as much as 17% within 2 years [47,48]. Bone protection agents, including bisphosphonates, monoclonal antibodies, and selective estrogen receptor modulators, are often used in conjunction with calcium and vitamin D supplementation, although calcium and vitamin D alone appear insufficient to halt ADT-related bone loss [36,46,47,48,49,50]. Vasomotor symptoms are commonly managed using hormonal therapies and non-hormonal agents [49].

In contrast, there are currently no standardized interventions specifically designed to prevent or reverse sarcopenia, obesity, SO, or the broader metabolic toxicities associated with ADT. Nonetheless, existing studies (Table 2) suggest that supervised exercise, including resistance training and nutritional counseling, can reduce fat mass and improve muscle mass and function in men treated with ADT [8,25,26,45,51,52,53,54,55,56]. In the IDEA-P trial, 32 PCa subjects on ADT were randomized to group-mediated exercise and nutritional intervention for a period of 12 weeks, resulting in improved muscle strength and mobility [24,25]. In a pilot trial (NCT04870515), researchers are evaluating diet and physical activity administered for 6 months, targeting PCa patients treated with ADT and radiation therapy, on changes in anthropometrics, metabolic abnormalities, and treatment outcomes. A trial (NCT03880422) evaluated the effectiveness of a nutrition and exercise intervention for 6 months to reduce ADT-induced obesity and frailty in PCa survivors. In 60 PCa patients, resistance exercise training for 20 weeks was observed to mitigate effects of ADT on body composition, muscle mass, strength, and aerobic capacity, with no additional benefits of protein supplementation [26]. An additional study is currently evaluating remotely monitored exercise interventions for 12 weeks in PCa patients on ADT (NCT06429813, unpublished). Similarly, a 12-week feasibility trial is evaluating behavioral exercise training for men undergoing ADT for PCa (NCT06250751, unpublished). A 6-month intervention combining aerobic and resistance exercise programs for 60 men on ADT demonstrated a significant and favorable effect on cardiorespiratory capacity, resting fat oxidation, glucose, and body composition [27]. A trial utilizing a 12-month internet-based lifestyle intervention (weight loss and resistance training) to eradicate obese frailty in PCa survivors (NCT06011499) is ongoing. Currently other early studies evaluating exercise regimens targeting other cancer patient populations have reported feasibility and promising results [57,58,59,60,61]. These efforts underscore growing recognition that exercise and nutrition interventions are both feasible and clinically relevant in this population.

However, most intervention trials in men receiving ADT have been limited by small sample sizes, short intervention durations, and limited assessment of muscle quality or metabolic biomarkers [8,25,26,27,45,51,52,53,54,55,56]. Many studies have lasted only 12–16 weeks, even though clinically meaningful adverse changes in body composition and metabolism often evolve over 6–12 months after ADT initiation [5,7,8,9,52]. Additionally, with the occurrence of multiple/related adverse effects with SO in PCa patients on ADT, it may be relevant to integrate multiple evidence-based interventions in parallel by blending and bundling evidence-based strategies [27] administered simultaneously to overcome the limitations of siloed interventions. Although the prevalence of obesity and sarcopenia have been reported, to date, inter-muscle fat infiltration, which is a cardinal feature of SO in PCa patients on ADT, has not been used as a biomarker nor as an outcome marker of an intervention. Importantly, there is a paucity of interventions that target the underlying mechanisms to mitigate the impact of ADT-induced SO and its effects on metabolic abnormalities, PCa, and all-cause mortality in this patient population. Most studies focus on body weight, lean mass, or strength, while largely overlooking muscle quality and myosteatosis. Yet, fatty infiltration of skeletal muscle may represent one of the most biologically and clinically informative features of ADT-induced sarcopenic obesity [11,14]. As a result, the true impact of ADT on skeletal muscle quality, and the potential reversibility of these changes through intervention, remains poorly understood.

### 3.4. Promising Approaches to Ameliorate SO and Other Metabolic Consequences of ADT

**Figure 3:**
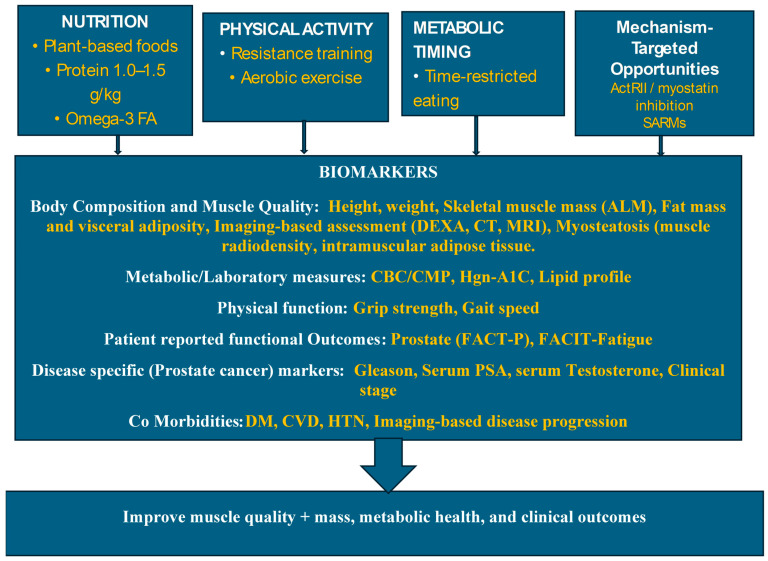
Proposed multimodal intervention framework targeting myosteatosis in men receiving androgen deprivation therapy (ADT). A multimodal intervention integrating resistance and aerobic exercise, dietary modification (including adequate protein intake and reduced intake of refined carbohydrates), omega-3 fatty acid supplementation, and metabolic timing strategies (e.g., time-restricted eating) is proposed to address key pathways underlying ADT-associated metabolic toxicity. These approaches collectively target systemic inflammation, insulin resistance, and dysregulated muscle remodeling, including pathways involved in lipid infiltration and impaired muscle regeneration. By acting on these interconnected mechanisms, such strategies may produce improvements in biomarkers of muscle quality, reduce intramuscular adipose accumulation, and mitigate the development of sarcopenic obesity in men receiving ADT, potentially leading to improved clinical outcomes.

A stronger intervention framework for men receiving ADT should be grounded in the biology of SO and myosteatosis. These observations suggest that interventions targeting inflammatory and muscle proteolysis pathways may be important [13,14,17,28,29,30,31,32,33,38,39,43,44,62,63,64,65,66,67,68,69,70,71,72] (Figure 3).

#### 3.4.1. Exercise

Exercise remains the most evidence-based cornerstone of intervention. Resistance training is particularly important for preserving or restoring muscle mass and strength, while aerobic exercise improves cardiorespiratory fitness, insulin sensitivity, and fat oxidation [16,41,42,43,44,45,46,47,48,49]. Systematic reviews and network meta-analyses support the benefit of exercise-based strategies for improving body composition in men with prostate cancer [54,55]. Given the multidimensional metabolic effects associated with ADT, combining resistance and aerobic exercise appears especially relevant.

#### 3.4.2. Protein and Micronutrient Support

Increased dietary protein intake in the range of 1.0–1.5 g/kg of body weight has been shown to stimulate muscle protein synthesis, and distribution of protein intake across meals may further enhance the anabolic response [73,74,75,76,77]. High-protein diets combined with resistance training may help preserve appendicular lean mass during weight loss [77]. Furthermore, combining high-protein, essential amino-acid-rich diets with increased fiber intake can foster a healthy, balanced gut microbiome (eubiosis) by promoting the production of beneficial short-chain and branched-chain fatty acids. Recent studies have explored consuming high levels of fiber and protein to increase specific gut bacteria that boost metabolic health, improve satiety, and support protein synthesis [78,79]. This strategy could serve as a novel dietary approach to manage sarcopenic obesity. Nonetheless, additional human studies employing advanced metabolomic techniques are necessary to thoroughly understand how macronutrients, particularly protein, interact with the microbiome. On the other hand, others have shown that higher protein intake, specifically from animal food sources, is protective against sarcopenia but is also linked to a higher obesity risk [80]. Thus, future human studies evaluating leaner protein sources in this target population with increased fiber intake should be evaluated.

Androgen deprivation therapy (ADT) significantly increases the risk of bone fractures in addition to loss of muscle in men, though it remains unclear if different types of ADT or varying schedules (continuous vs. intermittent) offer different risks for osteoporosis. ADT has also been shown to significantly increase osteoporosis-related fracture risk in prostate cancer patients. Besides direct treatment-induced bone loss, this risk is heightened by pre-existing factors such as older age, low bone mineral density (BMD) and increased falling, driven by muscle weakness, poor balance, and postural hypotension [81,82]. Vitamin D and calcium supplementation may also support muscle function and skeletal health, particularly in the setting of concomitant ADT-related bone loss [36,46,47,48,49,50,83]. Improving bone mineral density (BMD), decreasing the fracture rate, and improving muscle loss in these patients has not been explored in conjunction with interventions such as exercise, smoking abstinence, adequate calcium, protein, and vitamin D intake, along with the use of bisphosphonates or calcitonin.

#### 3.4.3. Omega-3 Fatty Acids

Dietary omega-3 fatty acids represent a promising strategy because of their anti-inflammatory and potential anti-catabolic effects. Experimental evidence has demonstrated that the ubiquitin–proteasome pathway, as indicated by upregulation of NF-kB, accounts for the majority of skeletal muscle degradation, stimulated by several proinflammatory cytokines including TNFα and Il-6 [62]. Pro-inflammatory cytokines (Il-6 and TNF) are upregulated by leptin and associated with truncal obesity, which has been shown to exacerbate sarcopenia [13,28,43,63]. Obesity has been shown to induce leptin resistance, promoting reduced muscle fatty oxidation and ectopic fat deposition impacting muscle quality, a unique feature of SO [11,67].

The deleterious effects of inflammatory cytokine-induced myogenesis was inhibited by ω-3 fatty acids-rich diets [84,85]. Oral administration of fish oil-derived ω-3-FA, EPA, and/or DHA have been shown to attenuate tumor growth, weight loss, and/or muscle wasting in animal models of cancer [86,87]. In a cell culture model of myogenesis, EPA suppresses increases in the activities of NF-κB, caspase 8, and proteasome in differentiating C2C12 myotubes induced by PIF or TNFα, thereby suppressing apoptosis and necrosis [88,89]. The deleterious effects of inflammatory cytokines on myogenesis have also been shown to be inhibited by EPA [53,89]. Several recent laboratory studies indicate that EPA may attenuate protein degradation by preventing NF-κB translocation to the nucleus [51,52]. In a recent meta-analysis (2024), supplementation of ω-3 FA had a favorable effect on improving lipid levels, reducing pro-inflammatory cytokines among patients with metabolic syndrome and cardio vascular disease [90], as well as improved clinical outcomes in cancer patients undergoing chemotherapy [91]. ω-3 FA has been shown to reduce proinflammatory cytokines even in cancer patient populations [92,93,94]. In a preliminary clinical trial [64] using ω-3 FA supplement-4 g Lovaza^®^ plus MA and nutritional supplements administered for 6 weeks to thirty-six (36) stage II-IV cancer patients, we observed stable anthropometrics, significant increase in serum albumin, and functional status with no toxicity. Proteasome activity was inhibited in nine out of 14 patients (64%) post treatment. Serum cytokines showed that both TNFα and IL-6 declined or remained stable with ω-3 FA. Our observations are provocative in that this improvement/stabilization of skeletal and visceral proteins and proinflammatory cytokines and improvement in functional status occurred after initial weight loss, diagnosis of cancer, and while on active cytotoxic therapies [64]. In our preliminary study, ω-3 fatty acid intake of all men diagnosed with PCa was significantly lower (75% lower) than the recommendations of the USRDA for optimal ω-3 fatty acids (11.2 g per week) [95]. Although direct trials with men receiving ADT are needed, omega-3 fatty acids may represent a feasible adjunctive intervention targeting inflammation and muscle catabolism for this population.

#### 3.4.4. Plant-Rich, Phytochemical-Based Dietary Strategies

We and others have shown that plant-based phytochemicals are promiscuous in their targeting and have been shown to target several critical hallmarks of carcinogenesis, without toxicities and intolerances at these doses [96,97,98,99,100,101]. Flavonoids are polyphenolic phytochemical compounds found in several plant foods including fruit, vegetables, and grains. Several in vitro, preclinical, and a few early randomized clinical trials have demonstrated that the effects of flavonoids are mediated by inhibiting the NF-κB pathway [102]. Genes regulated by the transcription factor NF-κB have been shown to modulate inflammation, cellular transformation, tumor cell survival, proliferation, invasion, angiogenesis, and metastasis. Phytochemicals containing flavonoids can suppress the proinflammatory cell signaling pathways [103]. Flavonoids such as quercetin, genistein, curcumin, indole-3 carbinol, sulforaphane, resveratrol, anthocyanins, and epigallocatechin 3-gallate regulate the gene expressions of several pro-inflammatory molecules such as NF-κB, and modulate the expression and activation of proinflammatory cytokines tumor necrosis factor-alpha (TNF-α), interleukin-6 (IL-6), and interleukin-8 (IL-8) [104,105,106,107,108]. Our team reported that EGCG potently and selectively inhibits the proteasome activity in intact human cells, leading to the accumulation of IkB-α and p27 proteins, resulting in growth arrest [109,110]. Our findings in preclinical models and early trials provide additional evidence for the safety and chemopreventive effect of GTC in preventing the progression of PCa with EGCG [111,112]. Phytochemicals found in pomegranate extract has been shown to target multiple signaling pathways specific to PCa, including STAT3 phosphorylation, NF-κB activation, inhibition of IGF-1/AKT/mTOR signaling [113], androgen biosynthesis enzymes such as 5α-reductase type I and 3β-hydroxysteroid dehydrogenase type II,. [114,115,116], YP1B enzyme activity/expression, and decreasing of serum PSA levels [117,118,119]. Other botanicals including Indole-3-carbinol [120,121], curcumin [122,123], and sulforaphane [117,124] target NF-κB. With the promising data evolving from laboratory and early clinical trials, the recommendation to increase plant foods rich in phytochemicals to prevent cancer and other metabolic disorders has been consistent worldwide. Although these studies have targeted men with or at high risk for prostate cancer, currently, there are no trials targeting men receiving ADT.

#### 3.4.5. Time-Restricted Eating and Fasting-Based Approaches

Periodic fasting (PF), intermittent fasting (IF), and time-restricted eating (TRE) have been used to refer to periods of fasting or fasting mimicking diets (FMD) that encompass eating patterns with little or no energy intake restriction over an extended period of time in a day. Studies of IF (e.g., 60% energy restriction on 2 days per week or every other day), PF (e.g., a 5 day diet providing 750–1100 kcal), and time-restricted feeding (TRF; limiting the daily period of food intake to 8–12 h or less/day) in normal, overweight subjects, and cancer patients have shown efficacy for weight loss and improvements in multiple health indicators including body composition, insulin resistance, and reductions in risk factors for cardiovascular disease [118,119]. Preclinical studies have consistently shown that FMD can significantly reduces markers of inflammation and obesity such as blood glucose, IGF-1, leptin, insulin levels, increases ketone bodies and IGFBP-1 levels, and ultimately reducing neoplasia incidence by 45%, which are [119,125]. Metabolic changes have been observed to remain even post-ending FMD and refeeding. Similar to animal models, subjects on fasting mimicking diets (FMD) have reported a 11.3% decrease in blood glucose level and a 24% reduction in circulating IGF-1 [125,126]. FMD reduced systolic BP, reduced body weight, waist circumference, total body fat, and trunk fat by 3%, and increased lean body mass. Studies in humans report a reduction in inflammatory markers with FMD [125,126]. Based on the concept of circadian rhythms, TRE is a daily eating pattern in which all nutritional intake occurs within a few hours (12 h or more) each day with no restriction of nutritional composition [119]. Time-restricted eating in humans for 2–6 months or more has demonstrated reduction in fat and improvement in markers of metabolic syndrome, cancer, cardiovascular, insulin-resistance, and diabetes [118]. Fasting periods in TRE and other fasting-based approaches have been shown to enable organisms to enter an alternate metabolic phase, which relies less on glucose and more on ketone body-like carbon sources [63]. TRE has been shown to be feasible and adoptable in adult populations, including in cancer patients, with no adverse effects [125,126,127,128,129]. TRE is especially appealing because it is pragmatic and may be more sustainable than prolonged fasting. However, in men receiving ADT, these strategies must be carefully evaluated to ensure that weight loss does not exacerbate muscle loss or worsen frailty.

#### 3.4.6. Emerging Pharmacologic Strategies

Beyond exercise and nutritional interventions, emerging pharmacologic strategies targeting muscle metabolism and anabolic signaling pathways are under active investigation. Inhibition of the activin receptor type II (ActRII) pathway [130,131] has shown promise in preclinical and early clinical studies, with agents such as bimagrumab [132] demonstrating the potential to increase lean mass and reduce fat mass. Similarly, selective androgen receptor modulators (SARMs) [133] are being explored as a means to preserve anabolic signaling in muscle while minimizing androgenic effects in prostate tissue.

Additional approaches include metabolic-targeting agents aimed at improving insulin sensitivity and mitochondrial function, as well as nutritional strategies incorporating high-protein supplementation, omega-3 fatty acids, and anti-inflammatory dietary patterns [134,135]. However, despite these advances, few interventions have specifically targeted muscle quality or incorporated imaging-based endpoints such as skeletal muscle radiodensity or intramuscular adipose tissue. The level of supporting evidence for each promising strategy discussed above is summarized in Table 3. This highlights a critical gap and underscores the need for mechanism-driven, multimodal interventions designed to address muscle mass and quality.

Collectively, these mechanisms support the concept that myosteatosis represents an active, biologically regulated phenotype driven by convergent endocrine, inflammatory, and metabolic pathways, rather than being a passive consequence of muscle loss alone.

## 4. Conclusions

ADT remains essential in the management of prostate cancer, but its benefits are accompanied by substantial adverse effects on body composition, metabolic health, physical function, and quality of life [5,6,7,8,9,10,52]. Among these, sarcopenic obesity represents a particularly important and underrecognized phenotype. Importantly, myosteatosis may be considered more than a body composition phenotype; rather, it represents a pathological alteration in skeletal muscle quality that integrates metabolic dysfunction, inflammatory signaling, and ectopic lipid deposition [11,12,14].

Although exercise- and nutrition-based interventions and emerging pharmacologic strategies have shown promise, the existing intervention literature is limited by short duration, modest sample size, and insufficient attention to biologic mechanisms and muscle quality [8,25,26,27,45,51,52,53,54,55,56]. Current approaches have also tended to be siloed rather than pragmatic and integrated, despite the fact that ADT produces multiple interconnected adverse effects. A more comprehensive strategy that combines evidence-based interventions in parallel may therefore be needed to address the complexity of ADT-induced sarcopenic obesity.

Recognizing myosteatosis as a central biomarker of sarcopenic obesity may help guide both risk stratification and targeted intervention strategies for men receiving androgen deprivation therapy.

## 5. Future Directions

Based on current evidence, future interventions designed to mitigate SO and associated metabolic abnormalities in men receiving ADT should evaluate comprehensive, bundled, evidence-based strategies initiated early in the course of treatment, ideally within 3 months of starting ADT. These bundled interventions should include: (a) a nutrition plan emphasizing low-glycemic, minimally processed foods, high intake of plant-based phytochemical-rich foods, and adequate protein intake; (b) omega-3 fatty acid supplementation and appropriate micronutrient support, including vitamin D and calcium when indicated; (c) structured aerobic exercise; (d) progressive resistance training; and (e) carefully monitored time-restricted eating or related metabolic timing strategies when feasible and safe. Future clinical trials should incorporate objective measures of muscle quality, including imaging-based assessment of myosteatosis using CT or MRI, as core biomarkers of intervention efficacy. Because myosteatosis reflects both metabolic dysfunction and inflammatory signaling within skeletal muscle, it may provide a more clinically meaningful endpoint than lean mass alone. Clinical trials should ideally extend for at least 12 months, reflecting the timeframe over which metabolic and body composition changes become most pronounced [5,7,8,9,52]. Key endpoints should include changes in muscle mass, muscle strength, muscle quality, fat mass, metabolic syndrome biomarkers, inflammatory mediators, functional performance, symptom burden, adherence, acceptability, and quality of life. Mechanistic endpoints evaluating NF-κB signaling and pro-inflammatory cytokines may clarify the biological pathways through which these interventions exert their effects. If validated in future trials, mechanism-driven multimodal interventions may provide an effective strategy to mitigate ADT-related sarcopenic obesity, improve metabolic health and functional outcomes, and ultimately enhance survivorship in men with prostate cancer.

## Figures and Tables

**Table 1:** Distinguishing myosteatosis, sarcopenia, and sarcopenic obesity in oncology.

Feature	Myosteatosis	Sarcopenia	Sarcopenic Obesity
Primary definition	Impaired muscle quality with increased intramuscular fat (lipid infiltration)	Loss of skeletal muscle mass and strength/function	Coexistence of reduced muscle mass/function with excess adiposity
Key characteristic	Reduced muscle radiodensity (CT) or increased intramuscular adipose tissue (IMAT)	Reduced skeletal muscle index (SMI) and/or strength (e.g., grip strength)	Low muscle mass/function in the setting of elevated fat mass or BMI
Underlying biology	Ectopic lipid accumulation, impaired muscle regeneration, inflammatory and metabolic dysregulation	Muscle protein breakdown, reduced anabolic signaling, aging or disease-related catabolism	Combined effects of muscle loss and adipose-driven metabolic dysfunction (e.g., insulin resistance, inflammation)
Measurement approaches	CT-based skeletal muscle radiodensity (Hounsfield Units), IMAT (CT/MRI)	CT or DEXA-derived muscle mass (SMI, ALM); functional measures (grip strength, gait speed)	Combination of body composition (DEXA/CT) and functional measures
Clinical relevance	May represent an early and distinct phenotype; associated with metabolic dysfunction, treatment toxicity, and survival outcomes	Associated with frailty, reduced physical function, increased morbidity and mortality	Associated with worse metabolic profile, functional decline, and adverse oncologic outcomes
Temporal pattern	May precede measurable loss of muscle mass	Often develops progressively with aging, cancer, or treatment	May develop concurrently with or following sarcopenia in the setting of weight gain or adiposity
Reversibility/targets	Potentially modifiable through metabolic and anti-inflammatory interventions, exercise, and nutritional strategies	Targeted through resistance exercise, protein intake, and anabolic support	Requires multimodal strategies targeting both muscle and adiposity
Relevance in ADT	Highly relevant; reflects metabolic and inflammatory effects of ADT on muscle quality	Common in ADT; reflects loss of lean mass under hypogonadal conditions	Frequently observed in ADT; driven by concurrent fat gain and muscle loss

**Table 2:** Intervention trials targeting body composition and metabolic outcomes in men receiving androgen deprivation therapy (ADT).

Study/Trial	Sample Size	Duration	Intervention Components	Outcomes Measured	Muscle Quality Assessment
IDEA-P Trial [24,25]	32	12 weeks	Group-mediated exercise + nutritional counseling	Muscle strength, mobility	Not assessed
Resistance Training Trial [26]	60	20 weeks	Resistance exercise ± protein supplementation	Muscle mass, strength, aerobic capacity, body composition	Not assessed
Combined Exercise Trial [27]	60	6 months	Aerobic + resistance exercise	Cardiorespiratory fitness, fat oxidation, glucose metabolism, body composition	Not assessed
NCT04870515	Ongoing	6 months	Diet + physical activity (with RT context)	Anthropometrics, metabolic markers, treatment outcomes	Not specified
NCT03880422	Ongoing	6 months	Nutrition + exercise intervention	Obese frailty, body composition	Not specified
NCT06429813	Ongoing	12 weeks	Remotely monitored exercise program	Feasibility, physical function	Not specified
NCT06250751	Ongoing	12 weeks	Behavioral exercise intervention	Feasibility, adherence, functional outcomes	Not specified
NCT06011499	Ongoing	12 months	Internet-based lifestyle intervention (weight loss + resistance training)	Body composition, frailty	Not specified

“Not assessed” indicates that muscle quality measures (e.g., skeletal muscle radiodensity or intramuscular adipose tissue) were not included as study endpoints. “Not specified” indicates that available trial descriptions did not report whether muscle quality metrics were assessed. Most interventions focused on body composition, physical function, or metabolic outcomes, with limited incorporation of combining imaging-based muscle quality measures and metabolic outcomes.

**Table 3:** Strength of current interventions.

Exercise	Strong
Protein intake	Moderate
Omega-3	Emerging
Phytochemicals	Preliminary
TRE	Experimental
Metabolic-targeting agents	Emerging
SARMS	Emerging
Modulation of ActRII	Preliminary

## Data Availability

No new data were created or analyzed in this study. Data sharing is not applicable to this article.

## Abbreviations

The following abbreviations are used in this manuscript:

SO	Sarcopenic obesity
MYO	Myosteatosis
CC	Cancer cachexia
BMD	Bone mineral density
PCa	Prostate cancer
IGF-1	Insulin-like Growth Factor 1
IGFBP-1	Insulin-like growth factor-binding protein 1
MTOR	Mammalian target of rapamycin
EGCG	Epigallocatechin gallate
PIF	Proteolytic inducing factor
TRE	Time-restricted eating
FMD	Fasting mimicking diet
TRF	Time-restricted feeding
Kcal	Kilocalories
BP	Blood pressure
EPA	Eicosapentaenoic acid
DHA	Docosahexaenoic acid
NF-κB	Nuclear factor kappa-light-chain-enhancer of activated B cells
TNFα	Tumor necrosis factor-alpha
Il	Interleukins
CT	Computer tomography
L3	Lumbar vertebrae 3
SMI	Skeletal muscle index (SMI)
SMD	Skeletal muscle radiodensity
ADT	Androgen deprivation therapy
SARMS	Selective androgen receptor modulators
GDF1	growth differentiation factor 11
ActRII	Activin receptor type II
FAP	Fibro-adipogenic progenitor
